# The IGNITE network: a model for genomic medicine implementation and research

**DOI:** 10.1186/s12920-015-0162-5

**Published:** 2016-01-05

**Authors:** Kristin Wiisanen Weitzel, Madeline Alexander, Barbara A. Bernhardt, Neil Calman, David J. Carey, Larisa H. Cavallari, Julie R. Field, Diane Hauser, Heather A. Junkins, Phillip A. Levin, Kenneth Levy, Ebony B. Madden, Teri A. Manolio, Jacqueline Odgis, Lori A. Orlando, Reed Pyeritz, R. Ryanne Wu, Alan R. Shuldiner, Erwin P. Bottinger, Joshua C. Denny, Paul R. Dexter, David A. Flockhart, Carol R. Horowitz, Julie A. Johnson, Stephen E. Kimmel, Mia A. Levy, Toni I. Pollin, Geoffrey S. Ginsburg, on behalf of the IGNITE Network

**Affiliations:** 1 Department of Pharmacotherapy and Translational Research and Center for Pharmacogenomics, University of Florida (UF) College of Pharmacy, Gainesville, FL USA; 2 Center for Therapeutic Effectiveness Research, Perelman School of Medicine, University of Pennsylvania, Philadelphia, PA USA; 3 Division of Translational Medicine and Human Genetics, Perelman School of Medicine, University of Pennsylvania, Philadelphia, PA USA; 4 Institute for Family Health, New York, NY USA; 5 Weis Center for Research, Geisinger Health System, Danville, PA USA; 6 Institute for Clinical and Translational Research, School of Medicine, Vanderbilt University, Nashville, TN USA; 7 Division of Genomic Medicine, National Human Genome Research Institute, National Institutes of Health, Bethesda, MD USA; 8 Bay West Endocrinology Associates and MODEL Clinical Research, Baltimore, MD USA; 9 Department of Medicine, Indiana University School of Medicine, Indiana, IN USA; 10 Division of General Internal Medicine, Department of Medicine, Duke University Medical Center, Durham, NC USA; 11 University of Maryland School of Medicine, Baltimore, MD USA; 12 Regeneron Pharmaceuticals, Inc., Tarrytown, NY USA; 13 The Charles Bronfman Institute for Personalized Medicine, Icahn School of Medicine at Mount Sinai, New York, NY USA; 14 Departments of Biomedical Informatics and Medicine, Vanderbilt University School of Medicine, Nashville, TN USA; 15 Division of General Internal Medicine, Department of Medicine, Vanderbilt University School of Medicine, Nashville, TN USA; 16 Department of Population Health Science and Policy, Icahn School of Medicine at Mount Sinai, New York, NY USA; 17 Center for Clinical Epidemiology and Biostatistics, Center for Therapeutic Effectiveness Research, Perelman School of Medicine, University of Pennsylvania, Philadelphia, PA USA; 18 Departments of Biomedical Informatics and Medicine, Division of Hematology and Oncology, Vanderbilt University School of Medicine, Nashville, TN USA; 19 Duke Center for Applied Genomics and Precision Medicine, Duke University Medical Center, 101 Science Dr, Rm 2111, CIEMAS Bldg, Durham, NC 27708 USA

**Keywords:** Precision medicine, Pharmacogenomics, Genomics, Personalized medicine, Clinical decision support, Electronic health record, Implementation

## Abstract

**Background:**

Patients, clinicians, researchers and payers are seeking to understand the value of using genomic information (as reflected by genotyping, sequencing, family history or other data) to inform clinical decision-making. However, challenges exist to widespread clinical implementation of genomic medicine, a prerequisite for developing evidence of its real-world utility.

**Methods:**

To address these challenges, the National Institutes of Health-funded IGNITE (Implementing GeNomics In pracTicE; www.ignite-genomics.org) Network, comprised of six projects and a coordinating center, was established in 2013 to support the development, investigation and dissemination of genomic medicine practice models that seamlessly integrate genomic data into the electronic health record and that deploy tools for point of care decision making. IGNITE site projects are aligned in their purpose of testing these models, but individual projects vary in scope and design, including exploring genetic markers for disease risk prediction and prevention, developing tools for using family history data, incorporating pharmacogenomic data into clinical care, refining disease diagnosis using sequence-based mutation discovery, and creating novel educational approaches.

**Results:**

This paper describes the IGNITE Network and member projects, including network structure, collaborative initiatives, clinical decision support strategies, methods for return of genomic test results, and educational initiatives for patients and providers. Clinical and outcomes data from individual sites and network-wide projects are anticipated to begin being published over the next few years.

**Conclusions:**

The IGNITE Network is an innovative series of projects and pilot demonstrations aiming to enhance translation of validated actionable genomic information into clinical settings and develop and use measures of outcome in response to genome-based clinical interventions using a pragmatic framework to provide early data and proofs of concept on the utility of these interventions. Through these efforts and collaboration with other stakeholders, IGNITE is poised to have a significant impact on the acceleration of genomic information into medical practice.

## Background

**Fig. 1:**
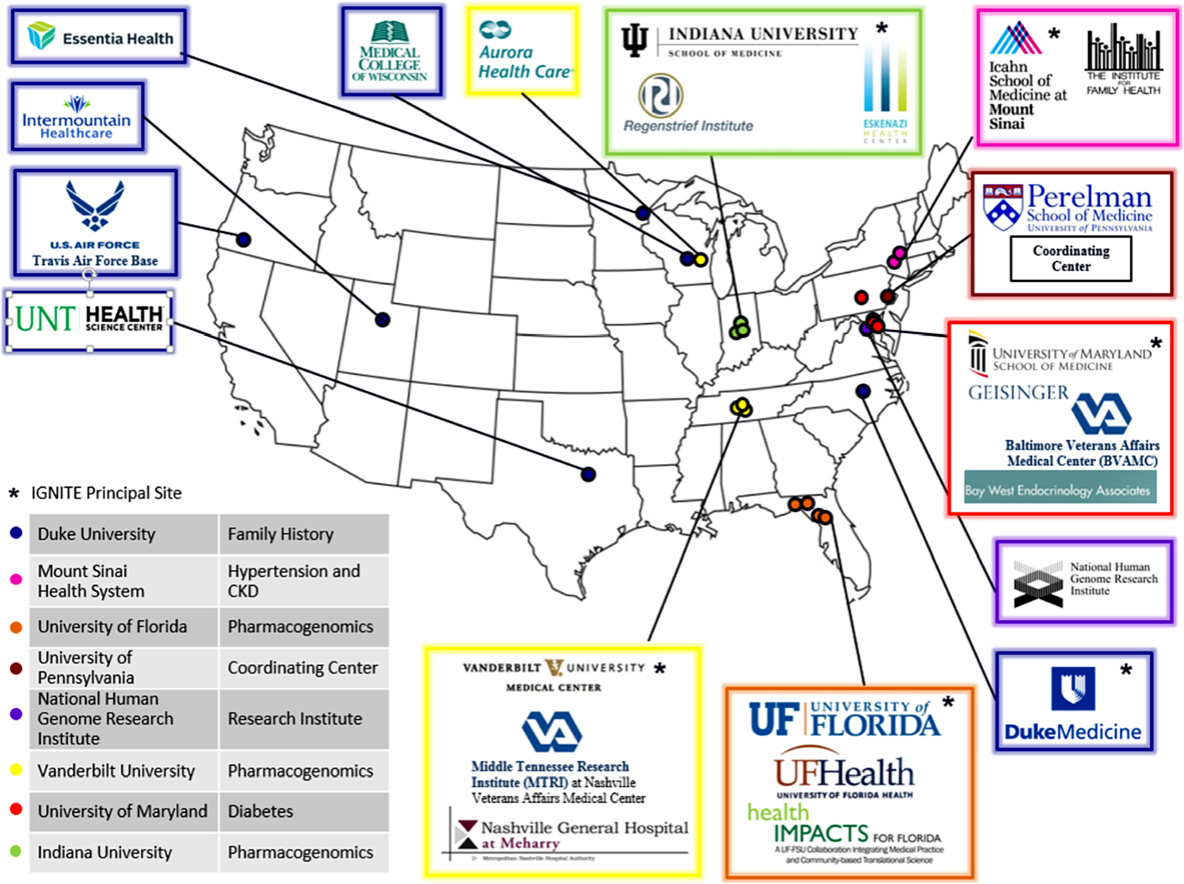
Legend: IGNITE Network Site Locations (Available at: http://www.genome.gov/27554264)

The potential benefits of genomic medicine, or the use of an individual patient’s genomic information (as reflected by family history, genotyping, sequencing or other DNA-based technology) into clinical decision making, are increasingly being recognized. Challenges to clinical implementation of genomic medicine have been identified at multiple levels including limited availability of evidence for clinical utility; ‘genomic unfamiliarity’ of providers, patients and families; limited access to advanced genetic testing and ambiguity of result interpretation; lack of reimbursement for genetic testing; and the need for real-time, point-of-care integration of test results with the electronic health record (EHR) and clinical decision support (CDS) tools. [8] Overcoming these challenges on a large scale will require collaborative efforts to develop effective health care delivery models of genomic medicine; demonstrate potential benefits of genomic data to improve patient outcomes and quality of care, and reduce costs of care; provide tools to support successful integration of genomic data in a platform-agnostic EHR environment; use CDS to facilitate streamlined and efficient care; and engage and educate providers, patients and payers in an efficient and effective manner. To support development and investigation of such practice models, the National Human Genome Research Institute (NHGRI) invited researchers to develop methods for, and evaluate feasibility of, incorporating an individual patient’s genomic findings into his or her clinical care. As a result, the IGNITE (Implementing GeNomics In pracTicE; www.ignite-genomics.org) Network was established with three initial sites and a coordinating center (CC) in 2013; three additional sites were added to the network one year later (Fig. 1).

## Methods

The goals of the IGNITE network are to 1) expand and link existing genomic medicine implementation efforts; 2) develop new collaborative projects and methods for genomic medicine implementation in diverse settings and populations; 3) contribute to the evidence base of outcomes following the use of genomic information for clinical care; and 4) define, share and disseminate the best practices of genomic medicine implementation, diffusion and sustainability in diverse settings. Each site participating in the NIH-funded IGNITE Network is developing and testing a clinical model for implementing genomic information (including diagnostic refinement, disease risk assessment, pharmacogenomic and family history data) into patient care with incorporation of the genomic information into the EHR and measures of outcomes in response to a genomic medicine intervention. In addition, sites are developing novel patient and provider educational models; testing CDS strategies to support clinical use of genomic data; utilizing novel dissemination and sustainability methods; and collecting data on factors influencing adoption of genomic medicine. All IGNITE projects are also examining implementation across a broad range of practice environments, including academic and non-academic settings as well as among diverse socio-economic and demographic patient populations.

### IGNITE network members

**Fig. 2:**
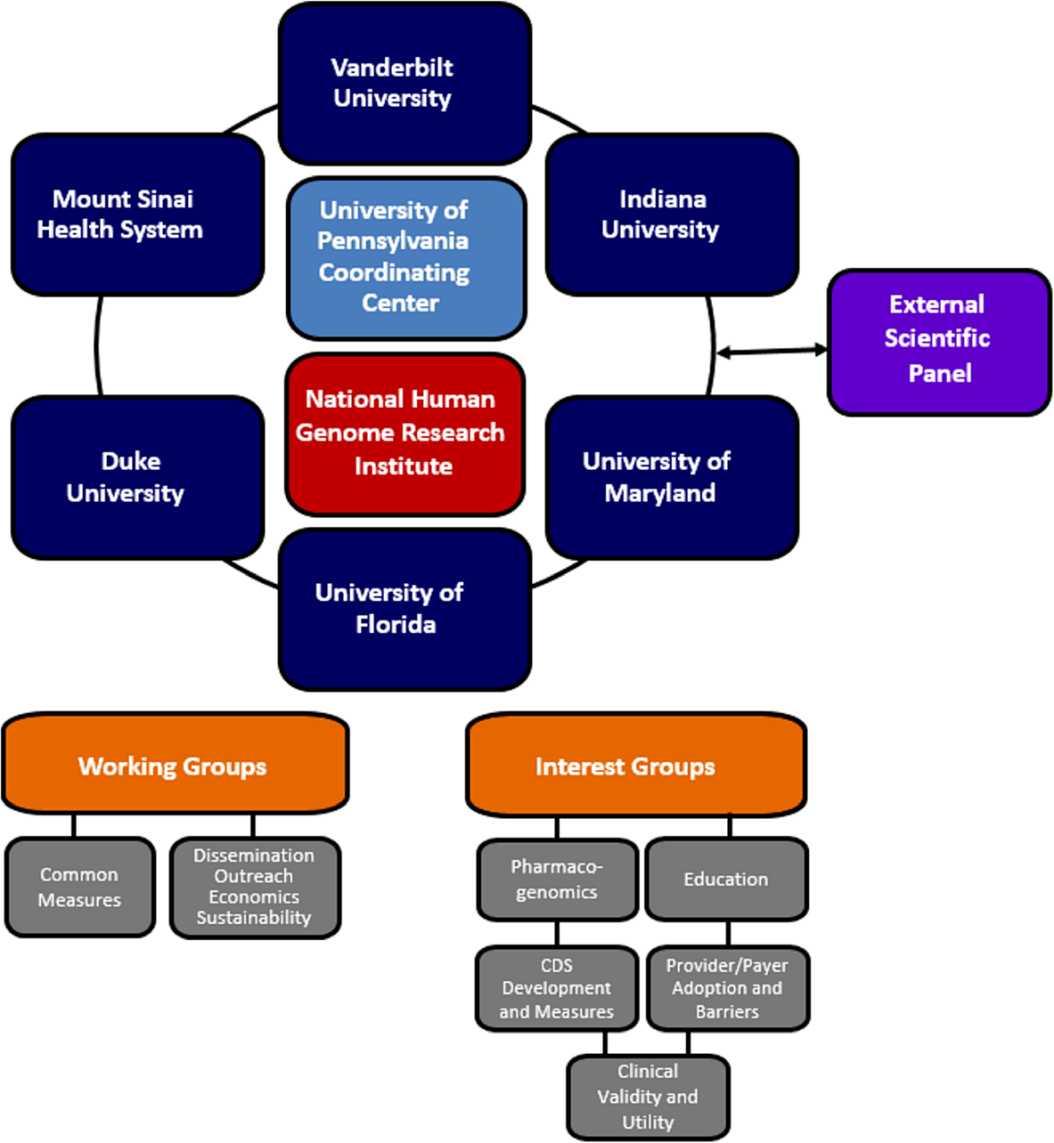
Legend: Organizational Structure of the IGNITE Network

The IGNITE Network consists of six member projects and a Coordinating Center which along with NHGRI program staff comprise the governing Network Steering Committee (Fig. 2). Each of the six IGNITE network projects are based in academic medical centers in partnership with a broad range of health systems with primary focus on health care delivery. Thus, the IGNITE Network is uniquely positioned to advance genomic medicine in a ‘real-world’ health care delivery framework with broad public health relevance. Although all IGNITE projects are aligned in their purpose of developing and testing clinical models for integrating genomic information into patient care, individual projects vary widely in their design and scope. Thus the results from these projects will form a broad foundation for implementation of genomic medicine. IGNITE members will not only advance the science of genomic medicine through their focused projects, but also by comparing strategies, sharing information and collaborating across projects (Table 1). Individual IGNITE projects are described below.

**Table 1:** Comparison of CDS, Return of Results and Educational Strategies for IGNITE Projects

Site/Project	Characteristics of CDS	Return of results	Educational strategies
Duke University: Implementation, Adoption and Utility of Family History in Diverse Care Settings	• Open source (OpenCDS) • Bidirectional • Based on HL7 Virtual Medical Record standard • Epic-based system	• Directly to patients and providers via CDS within the EHR	• English and Spanish language versions of FHH software • Printed and web based materials • Patient workbook and instructions for capturing FHH • Patient report for FHH results
Indiana University - INGenious: INdiana Genomics Implementation: an Opportunity for the UnderServed	• Eskenazi home-grown EHR system • Automated identification and randomization of patients • Capture of genetic variant data and reporting into EHR • Automatic alerts • Links to guidelines and supporting evidence for patients with pharmacogenomic results	• Directly to providers via CDS within the EHR	• Personal engagement with Eskenazi patient representative organization • Print materials in language-appropriate form in clinics
Icahn School of Medicine at Mount Sinai - Genetic testing to Understand and Address Renal Disease Disparities (GUARDD)	• Epic-based system that incorporates CLIPMERGE • Alert-based informed message for provider that integrates EHR and allele data • Based on HL7 standards • Integrated with Redcap • CDS alert includes link to provider and patient education materials	• Directly to providers via CDS within the EHR • To patients by research staff trained by genetic counselors • Genetic counselor available for consultation with patients or research staff	• Print materials (low-literacy, culturally appropriate, co-developed with community leaders and *APOL1*-positive patients) provided to patients at return of result and available for download by clinicians
University of Florida – UF Health Personalized Medicine Program	• Epic-based system • Alert-based informed message for provider that integrates EHR and allele data • Integrated with Redcap • CDS alert includes link to patient education materials	• Directly to providers via CDS within the EHR	• Print and online materials for patients and clinicians • Continuing education and academic courses for health care professionals and students
University of Maryland - Genomic Diagnosis and Personalized Therapy for Highly Penetrant Genetic Diabetes	• Epic-based system • Interface of screening tool and algorithm with EHR to produce alert for testing • Integration of actionable result report into EHR • Genetic result-based diagnosis/treatment recommendation alerts	• Direct communication of results to patients and entry into medical record • Customized materials provided to patients for communicating with other family members • If a variant of unknown significance is found, patients will be informed and invited to participate in additional research	• In-person throughout the study process (e.g., patient informed consent conducted by genetic counselor and research coordinator) • For patients with a pathogenic variant, study team and provider will discuss implications • Print materials also provided to patients throughout study
Vanderbilt University - Integrated, Individualized and Intelligent Prescribing (I^3^P) Network	• Multiple EHR systems (Epic, Veterans Affairs CPRS, McKesson, home-grown) • Bidirectional • Based on HL7 standards • Incorporates newly developed HL7 genomic data standard • Include interpretative recommendations • Link out to external information sources (e.g., MyCancerGenome.org) • Passive and active alerts	• Directly to providers via CDS within the EHR	• Print and online materials for patients and clinicians • Integrated with CDS • Provider focus groups

*CDS* clinical decision support, *HL7* health level-7, *EHR* electronic health record, *FHH* family health history, *CPRS* computerized patient record system

#### Duke University - implementation, adoption and utility of family history in diverse care settings

##### Background

Family health history (FHH) assessments have clearly been shown to identify persons at higher risk for common chronic disease, enabling preemptive and preventive steps, including lifestyle changes, health screenings, testing and early treatment as appropriate [5]. More recently Qureshi has shown prospectively the potential to identify presymptomatic individuals at elevated risk for common, chronic diseases and activate them to modify their risks [11] - an enormous opportunity to improve public health by implementing risk-based screening and prevention strategies. Yet, although FHH is a standard component of the medical interview and professional guidelines recommend screening strategies based upon FHH, its widespread adoption is hindered by three major barriers: (1) standard collection methods; (2) health care provider access to FHH information; and (3) clinical guidance for interpretation and use of FHH. The Duke University FHH project utilizes MeTree™, a platform that collects FHH directly from the patient and provides CDS to providers with guidelines-based recommendations for individuals at high risk for developing a common chronic disease.

##### Overview

This project utilizes a Genomic Medicine Model (GMM) developed in the context of the MeTree™ FHH pilot led by Duke University (https://precisionmedicine.duke.edu). The GMM is a novel flexible and adaptable model, with three components, designed to overcome barriers in the implementation of personalized medicine: 1) education of physicians and patients; 2) a health IT-based FHH platform, MeTree™, that collects FHH directly from patients on the front end and generates individualized risk-stratified evidence-based preventive care recommendations for physicians (as well as a pedigree and patient-oriented recommendations) on the back end, and 3) CDS to effectively interpret FHH information and carry out recommendations.

The IGNITE FHH project is a real-world intervention and outcomes study implemented in 28 primary care practices across 5 major health care delivery systems in the United States: Duke Health System, Medical College of Wisconsin, Essentia Health, University of NorthTexas Health Science Center, and the United States Air Force. Study sites include urban, suburban and rural settings serving a balance of minorities, women and socioeconomic statuses. Primary care practices at each site are assigned to use the MeTree™ FHH intervention and others, matched on practice demographics, perform usual care without it and serve as controls. Investigators are developing an open-source, standards-based CDS resource for FHH that will create EHR-enabled tools and CDS, with FHH data elements defined in the context of the HL7 Virtual Medical Record standard. This OpenCDS approach will provide a prototype for dissemination of evidence-based algorithms to EHRs to both patients and providers.

The goals of the Duke University FHH project are to 1) develop an optimal strategy for implementing MeTree into routine clinical practice in diverse settings; 2) demonstrate the effectiveness of MeTree in increasing uptake of risk-stratified evidence-based prevention guidelines; 3) create a standardized family health history storage database that can integrate with electronic medical records for bi-directional communication of family and personal history data and risk assessment results, and 4) disseminate guidelines for a FHH intervention in diverse practice settings.

##### Outcomes

The outcome measures for this project include patient, provider and system effects in the following domains: emotional (e.g., quality of life, satisfaction); behavioral (e.g., adherence, workflow processes); biological (e.g., demographics, FHH); clinical (e.g., laboratory data, patient population characteristics); and financial (e.g., socioeconomic status, medication costs). In addition, implementation measures include model reach, effectiveness, adoption, implementation integrity, implementation exposure and maintenance and sustainability of the intervention.

#### Icahn School of Medicine at Mount Sinai - Genetic Testing To Understand And Address Renal Disease Disparities (GUARDD)

##### Background

Chronic kidney disease (CKD) is commonly associated with hypertension (28 %) and affects 26 million American adults. Most cases of hypertension are managed by primary care providers. African ancestry populations with hypertension (HTN) have 2- to 3-fold higher risk of developing CKD, and a 5-fold increased risk to progress to end stage renal disease (ESRD) when compared with whites. HTN is a risk factor for progression of CKD and for increased cardiovascular disease (CVD) risk with CKD. Thus targeting blood pressure control as a modifiable risk factor may both reduce CVD in people with CKD and reduce progression of CKD to end stage disease. Recent discoveries demonstrate that testable alleles of the *APOL1* locus on chromosome 22 have a major effect on and explain most of the excess risk for hypertension associated CKD and its progression to ESRD in African ancestry populations. Hypertension-associated CKD in African Ancestry populations has emerged as a highly relevant and well-suited opportunity for a ‘prototype’ genomic medicine demonstration project for common chronic illnesses managed in primary care settings.

##### Overview

The GUARDD study is designed to generate essential insights for sustainable adoption and large-scale dissemination of genomic medicine in diverse clinical settings providing care for common adult-onset diseases in general, and for underserved African Ancestry populations with large excess burden of non-diabetic kidney diseases specifically. Investigators are using community-engaged approaches to enroll 2050 patients of African Ancestry at primary care facilities of the Mount Sinai Health System and of the Institute for Family Health, a network of community health centers with 13 sites in New York City. Patients must be 18–70 years of age, a patient at a participating care site, self-report African American/Black race and have a history of hypertension with no history of diabetes or kidney disease. Eligible patients will be randomized to an intervention or a control group in a seven-to-one ratio where *APOL1* genetic testing and return of results will be offered at the beginning or at the end of a 1-year evaluation period, respectively. The intervention group will be stratified naturally by *APOL1*-positive (increased genetic risk) and *APOL1*-negative (population-average genetic risk) genetic test results. Patients will be informed about their genetic risk status and its implications for their healthcare by trained study coordinators. Their providers will receive CDS stratified by *APOL1*-positive or -negative results in form of best practice alerts displayed at the beginning of encounters with enrolled patients. In addition to clinical follow-up data available through EHR data mining, standardized research data will be collected through clinical testing and questionnaires administered by study coordinators at 3 and 12-month follow-up visits.

##### Outcomes

Primary outcome measures will be compared among three arms of the GUARDD study, including *APOL1*-positive (estimated N ~ 250) and *APOL1*-negative (estimated N ~ 1550) intervention groups and the control group without intervention (estimated N ~ 250). Primary outcome measures include 1) change in systolic blood pressure and 2) utilization of blood and urine tests to assess kidney function in the *APOL1*-positive group compared with *APOL1*-negative group and control group at 3 and 12 months. Secondary endpoints will include comprehensive provider- and patient-oriented survey outcomes and process measures.

#### Indiana University - INGENIOUS: INdiana Genomics Implementation: an Opportunity for the UnderServed

##### Background

Genomic-based interventions such as pharmacogenomic testing have the potential to improve patient outcomes and reduce health care system costs. This could occur by reduction of adverse drug reactions and their associated medical costs, through the improvement of drug efficacy for expensive health care conditions, or by the targeting of effective, but expensive therapies to those most likely to benefit. Measurement of such costs is challenging, and not possible without an informatics infrastructure capable of measuring both financial costs and clinical outcomes. As a result, the business case for implementation of genomic and pharmacogenomics testing in large health care systems has not been persuasive.

##### Overview

Indiana University School of Medicine and the Indiana University Institute for Personalized Medicine in collaboration with the Eskenazi Health System are conducting a study to evaluate the economic and clinical outcomes associated with embedding a pharmacogenomics program in a system that serves as a health care safety-net in Indianapolis, Indiana. The INGenious project is a prospective, randomized, trial enrolling a total of 6000 patients, with 2000 patients assigned to a pharmacogenetic testing arm and 4000 to a control arm who will be followed, but not tested. A pharmacogenetic test, involving 51 SNPs in 16 genes will be carried out at the beginning of the study in patients in the testing arm upon prompting by an index medication: one of 24 selected as being supported by validated guidelines. This study is randomized between an intervention arm and one that receives no intervention in order that a genotyped group can be compared with one in which undisturbed, routine clinical care is carried out in patients taking the same drugs. Both arms will be followed for one year.

The specific aims of the INGENIOUS project include: 1) testing whether Clinical Laboratory Improvements Amendment (CLIA)-certified genotyping that is targeted at 24 widely used drugs is associated with significant reductions in hospital and outpatient economic costs incurred over 1 year; and 2) determining whether pharmacogenetic testing is associated with significant improvements in clinical outcomes over 1 year.

##### Outcomes

The INGENIOUS project is comparing the following outcomes between the two study arms: a) hospital and outpatient economic costs incurred over 1 year; and b) clinical outcomes over 1 year. Cost data are being obtained from patients randomized to either arm of the trial. The categories of inpatient and outpatient charges collected include medication, pharmacy, facility, laboratory test, treatment, professional and others. Clinical outcomes data being collected from the Eskenazi informatics system include the number of admissions, the number of emergency department visits, the number of clinic visits and returns to clinic. In addition, data are being collected on adverse drug reaction diagnostic codes and changes in drug regimens (according to prescribing patterns and the Medication Possession Ratio (MPR) of index medications).

#### University of Florida – UF Health Personalized Medicine Program

##### Background

There is substantial evidence that both efficacious and adverse responses of many drugs are significantly influenced by genetic variability. There are numerous examples of clinically actionable variants in pharmacogenetics and 10 % of all drugs contain pharmacogenetic information in their FDA-approved product label [14]. Nonetheless, there are limited examples of translation to practice in pharmacogenetics, making it an obvious starting point for implementation of genomic medicine.

##### Overview

The University of Florida (UF) Health Personalized Medicine Program (PMP, http://personalizedmedicine.ufhealth.org/) is a multidisciplinary clinical initiative to implement genomic medicine, with the program built around the long-term perspective that large amounts of genomic data are likely to be available on patients in the future. While the long-term goal of the PMP is to include disease risk prediction and defining disease prognosis, the initial focus is on implementing pharmacogenetics to guide drug decisions in clinical practice. [16].

The UF Health PMP IGNITE project builds on the existing framework for clinical pharmacogenomics already in place at UF Health, launched in 2011. Within this program, *CYP2C19*-guided clopidogrel therapy was initiated in 2012 and additional gene-drug pairs have subsequently been implemented clinically. [16]. UF Health PMP’s IGNITE project has 3 broad aims. The first aim is to expand and evaluate the clinical implementation of pharmacogenetic information to guide treatment decisions at UF Health. Specific drug-gene implementations are driven by the strength of the evidence, and potential impact on patient care in the specific patient population, with priorities set to some degree by interest/demand from clinicians within the health system. The second aim seeks to document that such a program can be successfully implemented outside a tertiary care university health system. This aim will focus on implementing in community practices and hospitals, with one of the goals being to identify the challenges that are common and unique between the UF Health implementations, and implementation in other setting. The third aim is focused on educational programs targeted at health sciences students, practicing clinicians and patients or the lay public. It is recognized that a significant barrier to implementation is knowledge of the area and comfort level among practicing clinicians with using genetic information to guide clinical decisions, and the innovative educational programs, which include the opportunity for the individual to receive personal genotype information to use during the educational activity, seek to help overcome these barriers.

##### Outcomes

Implementation metrics (e.g., test adoption rate, workflow processes, drug therapy changes initiated after test results) are being collected within and outside the UF Health system for all implementations. Clinical outcomes are being tracked either retrospectively (e.g., major adverse cardiovascular event rates in *CYP2C19*-clopidogrel patients) or prospectively (e.g., assessment of pain intensity, physical and emotional functioning with *CYP2D6*-codeine, tramadol patients). Knowledge, attitude and beliefs of educational program participants are also being assessed before and after completion of the educational activity.

#### University of Maryland - genomic diagnosis and personalized therapy for highly penetrant genetic diabetes

##### Background

Diabetes mellitus, a heterogeneous group of diseases characterized by hyperglycemia, affects over 25 million individuals in the United States and is a leading cause of cardiovascular disease, blindness, end-stage renal disease and death. Highly penetrant genetic forms of diabetes account for at least 1 % of diabetes, or over 250,000 cases nationwide. The most well-known and apparently most common highly penetrant genetic forms are traditionally known as “maturity onset diabetes of the young,” or MODY, which is most often caused by mutations in genes encoding pancreatic beta cell transcription factors or glucokinase. Several other syndromic and nonsyndromic forms of diabetes are caused by a single gene mutation. Distinguishing highly penetrant genetic forms of diabetes from the more common classes of diabetes, type 1 diabetes (T1DM) and type 2 diabetes (T2DM) often directly leads to more personalized and therefore more effective treatment, more accurate prediction of prognosis and familial risk assessment. Given these benefits, the American Diabetes Association (ADA) recommends that testing for monogenic diabetes be considered in several situations in children [1], but does not currently offer a specific algorithm for screening or testing and does not offer recommendations for screening adults.

##### Overview

This demonstration project will address the gap in monogenic diabetes detection and advance the implementation of genomic medicine in diabetes care by developing a real-world approach and EHR-compatible tools that can be disseminated broadly to improve screening, diagnosis and treatment of patients with highly penetrant genetic diabetes and their family members. In addition, the Personalized Diabetes Medicine Program (PDMP) will provide a model for finding and diagnosing highly penetrant genetic forms of other common diseases whose genetic architecture mirrors that of diabetes such as Alzheimer’s disease, Parkinson’s disease, cardiovascular disease and cancer.

First, this project will further develop the PDMP at the University of Maryland Center for Diabetes and Endocrinology and expand the Program into family medicine clinics and other partner centers (i.e., Baltimore Veterans Administration Medical Center, Geisinger Health System and Bay West Endocrinology Associates), and the larger community through extended site visits and media communications. The PDMP comprises an efficient screening tool to identify patients with diabetes who are strong candidates for having highly penetrant genetic forms of diabetes, a diagnosis algorithm that includes clinical and laboratory data, a novel highly penetrant genetic diabetes gene sequencing panel (40–55 genes), incorporation of genetic results into the electronic health record, and treatment recommendations customized to the genetic diagnosis.

This project also includes an evaluation of effects of implementing systematic screening and molecular diagnosis and treatment of highly penetrant forms of diabetes on clinical and patient-reported outcomes, resource utilization and barriers and facilitators of dissemination across diverse patient populations and healthcare delivery systems. Finally, a payer advisory panel is being engaged in the development of the impact evaluation process to enhance investigators ability to collect meaningful evidence to inform clinical practice recommendations and guide insurance coverage decisions.

##### Outcomes

The primary clinical outcome will be changes in HbA1c and blood glucose, with other clinical measures including changes in albuminuria, serum lipids, hypoglycemic episodes, weight change and visits to the clinic or emergency room. Effects of receiving or not receiving a molecular diagnosis and undergoing or not undergoing a treatment change will be evaluated. Data on multidimensional aspects of physical, mental and psychosocial aspects of health will be collected using validated instruments. Development of other patient-reported and patient-centered outcomes are being guided by the Payer Advisory Panel. Qualitative data regarding potential benefits and concerns raised by the intervention will also be collected as well as the potential impact of the intervention for at-risk family members.

#### Vanderbilt University - Integrated, Individualized and Intelligent Prescribing (I^3^P) Network

##### Background

The purpose of the I^3^P project is to perform a multisite test of the hypothesis that integrating genetic data within environments with diverse EHRs and informatics capabilities is feasible and can alter physician behavior toward a vision of individualized medicine. I^3^P is based on two, large-scale quality improvement initiatives for genome-based prescribing already in place at Vanderbilt, both launched in 2010. The Personalized Cancer Medicine Initiative (PCMI) provides routine, multiplexed tumor gene mutation testing for patients with various cancer diagnoses, including non-small cell lung cancer (NSCLC) and melanoma.[12, 13] The Pharmacogenomic Resource for Enhanced Decisions In Care and Treatment (PREDICT) program provides the organizational framework, technical infrastructure and institutional processes to allow clinically useful germline genotypes to be deposited into the Vanderbilt EHR and used in clinical care through electronic decision support.[10] Opportunities for genome-guided care are common – among the first 10,000 patients in PREDICT, 91 % of European ancestry individuals and 96 % of African ancestry individuals had actionably variants among five drug-genome interactions implemented in PREDICT, and the multiplexed genetic testing model used in PREDICT resulted in 35 % fewer tests than a traditional single drug-gene pair models.[15] For both PREDICT and PCMI, structured, computable variant data are decoupled from the interpretation of their clinical significance.

##### Overview

I^3^P will utilize existing, consensus-based pharmacogenomic knowledge in the creation of new technologies which provide shared clinical decision support to disparate external systems. Specifically, this project leverages the knowledge gained from PREDICT and PCMI to incorporate germline and somatic genomic testing and CDS at four external sites: Nashville Veterans Affairs Medical Center (NVAMC); Nashville General Hospital/Meharry Medical College (MMC); Aurora Health Care (AHC); and at Sanford Health (SH). The healthcare systems selected for this pilot effort, which use three different EHRs, include underserved minority and military populations and a community health system. NVAMC utilizes the Computerized Patient Record System (CPRS), which is in place at 152 VA medical centers and covers 8 million veterans. AHC and SH use Epic across 15 hospitals. MMC primarily serves an underserved population and employs different EHR systems for the inpatient and outpatient environments, requiring integration across both. Thus, successful implementation of genetic data and decision support modules will provide a model for translation to a wide variety of healthcare systems with diverse EHR ecosystems and informatics capabilities. Indeed, SH has already implemented CDS for several germline drug-genome interactions [7].

Three germline drug-gene pairs are being targeted for I^3^P implementation: *CYP2C19*-clopidogrel, *SLCO1B1*-simvastatin and *VKORC1/CYP2C9*-warfarin. In oncology, genetic testing and decision support is being implemented for erlotinib in the treatment of NSCLC and vemurafenib for melanoma. Initial implementation will focus on clopidogrel and erlotinib.

##### Outcomes

The primary outcomes will be rates of genome-tailored prescriptions at each site. As with the UF Health IGNITE program, we will seek to evaluate antiplatelet prescriptions based on *CYP2C19* metabolizer status. Retrospective evaluations will seek rates of major adverse cardiac events based on genotype and prescription status using automated electronic phenotype algorithms applied to EHR data [4]. Similar evaluations of genome-tailored prescriptions and dosage evaluation will be undertaken for each drug-genome interaction implemented. Implementation challenges vary based on sites differences: a government-run VA, two large non-academic health systems, and a university paired with a local county hospital. Additionally, we are finding that each EHR system poses unique challenges to incorporation of genetic information and sending a CDS alert. We will assess provider attitudes, identifying key road blocks and processes for overcoming these challenges, and develop technologies to facilitate incorporation of computable representations of genomic knowledge into diverse EHRs.

#### Coordinating Center (CC) – University of Pennsylvania

The goal of the IGNITE CC is to support the success of individual sites and stimulate collaboration across projects to produce and disseminate generalizable knowledge. Such knowledge can be used to sustain and expand existing efforts and to inform implementation in other clinical settings.

To accomplish this goal, the CC collaborates with study investigators and the NHGRI to coordinate, facilitate and support program activities at multiple levels, including handling network administrative logistics (e.g., organizing meetings and conference calls), developing and maintaining the IGNITE website, and providing mechanisms for internal communications among projects and with NHGRI. CC faculty co-investigators contribute their expertise to the IGNITE projects in the areas of medical genetics, genetic counseling; statistical genetics; clinical decision support tools; bioinformatics; outcomes measurement; epidemiology; clinical trials; and ethical, legal and social issues in genomics. They work with IGNITE site investigators to address challenges, apply solutions and conduct and facilitate cross-study analyses in order to maximize the public health impact of these genomic implementation studies.

The CC team uses the Consolidated Framework for Implementation Research (CFIR; Damschroder et al. 2009) to assist sites in informing the development of their implementation interventions, assessing the effectiveness of these implementations, and improving their chances of success, sustainability and dissemination. CFIR provides an overarching and flexible typology that can be applied to implementations to learn what works and why across multiple contexts. The use of a mutually agreed-upon framework helps guide each step of the implementation process, e.g., identifying factors that promote or impede implementation, integrating genomic findings into electronic health records, developing clinical decision support tools, and defining and measuring outcomes of implementation

The specific aims of the CC thus are to: 1) support IGNITE projects through logistics planning, maintenance of continuous dialogue and sharing across projects and with NHGRI, identification of barriers and their solutions, and dissemination of these solutions across all sites and to the broader scientific community; and 2) stimulate and leverage synergy across IGNITE and with other initiatives to develop common themes of implementation, optimize return of results, develop and disseminate detailed process and outcome measures, create new generalizable knowledge, evaluate the efficacy and effectiveness of implementation strategies, and develop methods to sustain and expand the implementation of genomics into clinical practice.

### Network organization and governance

The IGNITE Network is organized into an executive committee (Coordinating Center principal investigator, Steering Committee Chair and NHGRI program staff) and steering committee (IGNITE principal investigators and NHGRI program staff), that along with their co-investigators, meet by conference call monthly and in-person three times annually (Fig. 2). Leadership of the steering committee rotates through principal investigators, who each serve a one-year term. The Coordinating Center is responsible for network-wide communications and data and project management. Two working groups spearheaded the initial network-wide projects and data sharing initiatives: 1) the Common Measures Working Group and 2) the Dissemination, Outreach, Economics and Sustainability Working Group. Other network-wide interest groups have subsequently been developed, including: 1) Pharmacogenomics; 2) Education; 3) CDS Development and Measures; 4) Provider and Payer Adoption and Barriers; and 5) Clinical Validity and Utility interest groups. A six-member External Scientific Panel (ESP) provides independent input to NHGRI bi-annually about network progress and direction.

### Data collection

The IGNITE network is collecting various types of data: clinical, family history, genetic and outcomes (Table 2). Genomic data include individual patient genetic and pharmacogenetic test results and family health history data, including pedigree and personal risk assessment reporting. Genetic testing that is being reported to providers and patients is being conducted in College of American Pathologists–Clinical Laboratory Improvement Amendments (CAP–CLIA) certified clinical laboratories (University of Florida, Indiana University, University of Maryland, Mount Sinai and Vanderbilt); if DNA samples are retained for further research purposes, this is done in the research institution’s secured biobank or biorepository facility. Family health history data are maintained in a secure cloud server behind the Duke University firewall. Genetic and pharmacogenetic test results, risk assessment reports based on family history and clinical data, and health pedigrees are returned to the clinician and/or patient via the EHR in the clinical process of care. Although the clinical and patient outcomes measured vary by project, all IGNITE sites anticipate that the patient’s clinician will use the data that are returned to inform patient care decisions, per the IRB-approved protocols. The goals of IGNITE are to determine whether these data improve patient-specific outcomes, such as decreased adverse effects with application of pharmacogenetics test results, lower blood pressure with patient knowledge of *APOL1* genetic risk status, safer and more effective glucose control or reduced glucose monitoring based on specific diabetes subtype diagnosis, or improved preventive care and screening based on individual knowledge of family health history.

**Table 2:** IGNITE network strategies for data collection, distribution and use in patient care

	University of Florida	University of Maryland	Indiana University	Vanderbilt University	Duke University	Icahn School of Medicine at Mt. Sinai
Type of Genomic Data Collected	Multiple pharmacogenomic variants^a^	Pathogenic/likely pathogenic variants in monogenic diabetes genes	Multiple pharmacogenomic variants^a^	Multiple germline and somatic pharmacogenomic variants^a^	Family health history pedigree and personal risk assessment report	Test for variants of *APOL1* gene that increase kidney failure risk in adults of African ancestry
Sample/Data Collection Method^b^	Blood or sputum, QuantStudio, Luminex xTAG, GenMark or ViiA 7	Blood, Ion Torrent, Sanger Sequencing	Blood or sputum, QuantStudio	Blood, Illumina-ADME array; transitioning to QuantStudio for future testing	Patient enters data into web-based data collection ool	Blood or sputum
						TaqMan PCR
Sample/Data Storage and Security^c^	Clinical data in EHR; research data/samples in biorepository/IDR; secure facilities	DNA in secure freezer; data in binary (.BAM) and VCF files, text, spreadsheets, chromatograms, in secure software	DNA secured via limited access room and locked freezers; data in secured database and Eskanzi EHR	Data stored on individual site servers; Veterans Affairs site data on FISMA compliant server	Cloud server/risk assessment report and health pedigree in patient EHR; secured server	Clinical data in EHR; secured server
Test Results and/or Data Distribution to Providers or Patients^b^	Via EHR as lab results and CDS in EHR to providers, and/or secured communication to provider with clinical guidance	Clinical consult note in EHR, patient provided custom report, consult note, letters for patient and family members	Via EHR for physician; samples available upon request from biobank	Identifiable data integratedinto EHR for clinical decision making.	Via EHR (provider report); via web-based tool (patient report)	Through CDS in EHR to primary care clinicians; in person and in writing to patients
Use of Genomic Information in Process of Care	CDS alert and/or PGx consult used to inform drug therapy changes	Results may change diagnosis (to MODY or other monogenic diabetes type), treatment plan or follow up frequency	Results used to help guide patient care and therapy choices	CDS alert at order entry will indicate drug therapy alternative (active CDS) or PGx consultant will send message to provider (passive CDS).	Risk assessment report of elevated familial risk based on guidelines for a finite number of conditions and diseases given to providers/patients	CDS alerts to providers to help risk stratify hypertension patients; low-literacy materials to patients to guide care choices, activation and adherence
Expected Impact on Clinical Decision Making	Optimized drug therapy decision making with incorporation of genetic information in clinical decision making process	Potential change in treatment modality	Improved therapy decision making as a result of patient-specific genetic information	Changes in drug prescribing in individuals with SNPs that indicate lack of efficacy or increased toxicity.	Improved FHH in primary care; enhanced adherence to guidelines; promotion of patient-provider communication	Increased attention to blood pressure control and renal disease screening for clinicians and patients, improved patient-clinician communication
Potential Benefit to Patient	Optimal drug therapy selection for improved efficacy and/or safety and reduced risk of adverse outcomes	Optimal, cost effective, glucose control; provision of more accurate diabetes risk assessment and diagnosis	Optimal drug therapy selection for improved efficacy and/or safety and reduced risk of adverse outcomes	Optimal drug therapy selection for improved efficacy and/or safety and reduced risk of adverse outcomes	Education on FHH collection; improved patient-provider communication; improved preventive care/screeningbased on FHH	Better quality of care, improved knowledge/health behaviors, lower blood pressure, improved renal surveillance, better health outcomes and quality of life.

*CAP* College of American Pathologists, *CLIA* clinical laboratory improvement amendment, *EHR* electronic health record, *HIPAA* health insurance portability and accountability act, *FISMA* Federal Information Security Management Act of 2002, *IDR* integrated data repository, *CDS* clinical decision support, *PGx* pharmacogenetics, *FHH* family health history, *VCF* variant calling format

^a^Pharmacogenomic variants tested include germline and/or somatic testing of multiple clinically relevant single nucleotide polymorphisms (e.g., *CYP2D6*, *CYP2C19*, *TPMT*, *IL28B* [*IFNL3*], *CYP2C9*, *VKORC1*, *SLCO1B1*, *ABCC4*, *CYP2B6*, *CYP3A4/5*, *CYP4F2*, *DPYD*, *G6PD*, *HLA-B*, ITPA)

^b^Clinical data/samples are collected, stored and processed according to appropriate clinical compliance and/or security standards (e.g., CAP-CLIA accredited laboratory, HIPAA-compliant server) for all sites

^c^De-identified genomic data also deposited into the database of Genotypes and Phenotypes (dbGaP) when appropriate

## Results

Clinical and outcomes data from individual sites are anticipated to begin being published over the next two years, and in some cases, cross-network findings are being assembled. For example, the outcomes data from University of Florida’s clopidogrel-*CYP2C19* implementation are being presented in abstract form (Weitzel K, personal communication) and have led to a network-wide project on outcomes associated with clinical implementation of *CYP2C19*-guided dual antiplatelet therapy.

Similarly, individual sites are sharing with one another the critical challenges in implementation of genomics, which is leading to opportunities both to advance the field as a whole and to facilitate the groups learning from one another. These broad challenges to clinical implementation range from limitations in genotyping and EHR capabilities to a wide variety of educational needs as well as the development of practical strategies that are applicable to diverse practice settings.

Many of the initial challenges that have been encountered broadly in clinical implementation have resulted from real-world limitations in standardizing genotyping platforms for clinical practice, storing genetic data in computable formats, developing CDS capabilities, adapting clinical strategies as evidence and technology rapidly develop, and accessing outcomes data. IGNITE investigators are working collaboratively with external partners and IGNITE affiliate members to develop real-world, scalable solutions to these challenges that are informed by a broad sampling of clinical practice settings. For example, the IGNITE Pharmacogenetics Interest Group is partnering with in- and out-of-network sites to aggregate and analyze outcomes data on the clinical utility of pharmacogenetic testing for specific gene-drug pairs. The CDS Interest Group is collaborating with eMERGE (https://emerge.mc.vanderbilt.edu/) and other stakeholders to develop an online CDS resource (http://CDSKB.org) that will support clinical use of genomic data and its integration into the EHR. The Clinical Validity and Utility Interest Group is working to assess and document the clinical validity and clinical utility and economic implications of the interventions to ensure the sustainability of moving genomic medicine into clinical practice.

Other barriers to implementation that have been identified as being common to all IGNITE sites include gaps in provider knowledge and education, limited clinician experience with genetic testing and return of results, the need for user-friendly practice-based resources, and the need to educate widely variable target audiences. IGNITE interest and working groups, including the Education, Provider Adoption Barriers and Common Measures, are developing tools to identify and assess adoption barriers and are collaborating with other stakeholders (e.g., Inter-Society Coordinating Committee for Practitioner Education in Genomics, Genetics and Genomics Competency Center, eMERGE) to create and disseminate easily-accessible genomic medicine educational content, resources and strategies.

These and other collaboratively-developed resources supporting the use of genomic and pharmacogenomic data to guide patient care are being made available in an online “genomic medicine implementation toolkit” that will be hosted on the IGNITE website in early 2016.

## Discussion

### Goals for collaborative research and the future of genomic medicine

The IGNITE Network is poised to have a significant impact on the acceleration of genomic information into medical practice. The seven participating organizations include significant expertise in translational research and also have important linkages to health care delivery systems, and patient and advocacy groups. In the short term, each of the projects is defining novel implementation strategies that will eventually inform the broader clinical community on how to bring genomic tools into clinical workflows and health care. Specifically, IGNITE will provide the standardized methods for implementation as well as the common measures to assess the impact of implementation at the patient, provider and health system level (e.g., satisfaction, feeling of well-being, clinical outcomes, ability to help family members). The network will also communicate the results to a broad stakeholder community including regulators, payers and patient and community groups. Because IGNITE projects involve diverse settings, it will be possible to identify specific modifications needed to bring these innovative tools to underserved populations and a variety of practice venues serving diverse populations. Through affiliate members, IGNITE aspires to broaden the range and repertoire of genomic interventions and implementation strategies. Through its interactions with other NHGRI networks that are working with genomic tools and health care information and EHRs (such as the eMERGE [Electronic Medical Records & Genomics; https://emerge-network.org] Network and the CSER [Clinical Sequencing Exploratory Research; https://cser-consortium.org/] program), IGNITE will also provide synergies to improve the application of genomics to health care [2, 9]. In the long term, IGNITE seeks to become a knowledge hub on implementation of genomic medicine in the real world. It also aspires to deliver a tool box and know how – based on the Network’s collective wisdom and insights – that others can use to integrate evidence-based genomic applications into healthcare. With the recent announcement of the United States’ Precision Medicine Initiative (wh.gov/precision-medicine, http://www.nih.gov/precisionmedicine/), IGNITE can play a strategic role in assisting this effort to deliver on its goal to impact populations and eventually population health (including educating providers and the general public about precision medicine).

## Conclusion

The IGNITE Network is a novel innovative collaborative series of projects aiming to enhance the translation of validated actionable genomic information into clinical settings and thus create a road map for implementation with broad potential for use. IGNITE is also a series of pilot demonstration projects that aim to develop and use measures of outcome in response to genome-based clinical interventions using a pragmatic framework to provide early data and proofs of concept on the utility of these interventions. IGNITE fills a critical gap in the translational genomics continuum in T3 (translation to practice) [6] research and will define for many earlier stage genomic research projects a methodology and pathway for moving these findings into clinical medicine. With the advent of the Precision Medicine Initiative as a national agenda, IGNITE’s mission and activities are timely and should provide important guidance to the research and clinical community to enable more precise genome-informed medical care.

## Abbreviations

ADA: American Diabetes Association
AHC: Aurora Health Care
CAP: College of American Pathologists
CC: coordinating center
CDS: clinical decision support
CFIR: consolidated framework for implementation research
CKD: chronic kidney disease
CLIA: clinical laboratory improvements amendment
CPRS: computerized patient record system
CSER: clinical sequencing exploratory research
CVD: cardiovascular disease
EHR: electronic health record
eMERGE: electronic medical records and genomics
ESRD: end stage renal disease
FDA: food and drug administration
FHH: family health history
GMM: genomic medicine model
GUARDD: genetic testing to understand and address renal disease disparities
HTN: hypertension
IGNITE: implementing genomics in practice
INGENIOUS: INdiana Genomics Implementation: an Opportunity for the Under Served
IRB: Institutional Review Board
MMC: Nashville General Hospital/Meharry Medical College
MODY: maturity onset diabetes of the young
MPR: medication possession ratio
NHGRI: National Human Genome Research Institute
NIH: National Institutes of Health
NSCLC: non-small cell lung cancer
NVAMC: Nashville Veterans Affairs Medical Center
PCMI: personalized cancer medicine initiative
PDMP: personalized diabetes medicine program
PMP: personalized medicine program
PREDICT: pharmacogenomic resource for enhanced decisions in care and treatment
SH: Sanford Health
T1DM: type 1 diabetes
T2DM: type 2 diabetes
UF: University of Florida
